# Complete horizontal gaze palsy due to bilateral paramedian pontine reticular formation involvement as a presentation of multiple sclerosis: a case report

**DOI:** 10.1186/s12883-019-1494-0

**Published:** 2019-10-27

**Authors:** Amirhossein Akhavan Sigari, Masoud Etemadifar, Mehri Salari

**Affiliations:** 1 Alzahra Research Institute, Isfahan University of Medical Sciences, Isfahan, Iran; 2 Department of Neurology, Alzahra University Hospital, Isfahan, Iran; 3 Department of Neurology, Shahid Beheshti University of Medical Sciences, Tehran, Iran

**Keywords:** Multiple Sclerosis, Paramedian Pontine Reticular Formation, Bilateral Gaze Palsy, Ocular Movement Disturbance, Magnetic Resonance Imaging

## Abstract

**Background:**

Demyelinating central nervous system diseases include several disorders that multiple sclerosis (MS) is identified as the most common among them. Ocular movement disturbances are a typical presentation in MS patients where lesions affect the complex and interconnected pathways involved in eye motion. Centers for gaze control are located in the pons primarily; therefore, lesions involving these centers can be presented with abnormalities in gaze. However, bilateral lesions in pontine gaze centers are exceptionally rare.

**Case presentation:**

A 16-year-old girl with bilateral horizontal gaze palsy was referred to the neurology clinic. Magnetic resonance imaging of the patient indicated bilateral hyperintensities in the pons at the level of the paramedian pontine reticular formation. The patient was diagnosed with multiple sclerosis with respect to clinical and imaging findings and managed.

**Conclusion:**

Ocular movement abnormalities are a commonly encountered manifestation in patients with multiple sclerosis, however, bilateral gaze palsy is an exceptionally rare sign and should guide the physician to contemplate for anticipated lesions in the pons, and suspect MS, especially in childbearing-aged women. Although an extensive workup should also be done to rule out possible mimickers.

## Background

Multiple sclerosis (MS), known as a chronic inflammatory disease primarily involving the central nervous system, can be presented with various clinical manifestations such as sensory problems, visual disturbances, weakness, gaze abnormalities, etc. with some being more common and others less.

Impairment of eye movements is a common feature of MS and up to three quarter of patients with this disease experience eye movement abnormalities sometimes during their disease course [1]. In terms of the location of MS lesions in the brain, brainstem, cerebellum, and the pathways affected, ocular movement abnormalities can demonstrate diverse clinical presentations. Posterior fossa lesions in magnetic resonance imaging (MRI) of MS patients typically involve the cerebellum, middle cerebellar peduncles and those areas adjacent to the fourth ventricle [2].

Although specific ocular movement abnormalities, such as nystagmus and internuclear ophthalmoplegia (INO) are more frequent in MS, bilateral horizontal gaze palsy is a rare clinical manifestation [3]. Accordingly, we will discuss a case who was presented with complete impairment of horizontal gaze. The disease course and MRI findings of the patient resulted in the definite diagnosis of MS with respect to the 2017 revised McDonald’s criteria, and the origin of the patient’s eye problems was linked to lesions located in the pons at the level of the paramedian pontine reticular formation (PPRF).

## Case presentation

A 16-year-old Iranian girl was referred to the neurology department because of having bilateral horizontal gaze palsy. The gaze palsy was sudden in onset, developing overnight. The patient also reported a history of paresthesia in both of her feet since 2 months ago, which had progressed in the course duration of 2 weeks reaching her trunk at the level of the umbilicus. She experienced similar symptoms in both of her hands approximately 1 year ago. Regarding, her past medical history was otherwise normal. She used no medications and denied using illicit drugs, tobacco and alcohol.

Considering physical examination, the patient had complete bilateral horizontal gaze palsy in the horizontal plane while vertical gaze was normal, aside from slightly decreased velocity of saccadic movements. Pupils were midsize and reactive, and nystagmus, diplopia or blurry vision were not found. Mild bilateral peripheral facial nerve palsy was also presented in the physical exam. Hyperreflexia in all four limbs was detectable without clonus, plantar reflexes were down going, and muscle forces were not reduced. There were no apparent signs of spasticity and ataxia. All other physical exam findings were normal.

**Fig. 1:**
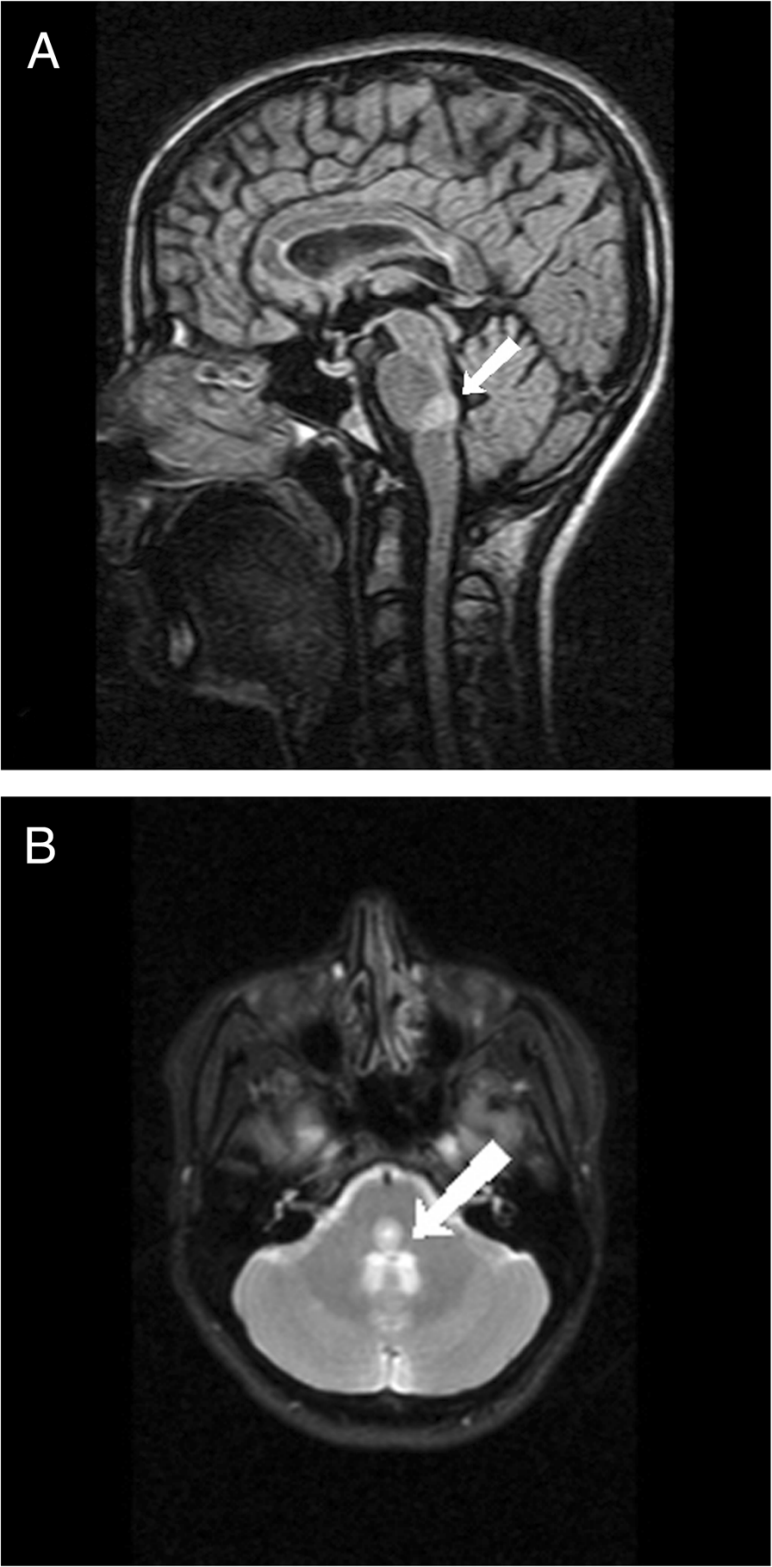
**a** Sagittal FLAIR MRI showing a hyperintense lesion (arrow) in the PPRF area. **b** Axial T2-weighted MRI showing bilateral hyperintensities (arrow) in the PPRF area in the pons at the approximate level of the abducens nucleus and facial colliculus

Magnetic resonance imaging indicated multiple hyperintense subcortical, and periventricular lesions visible on the fluid-attenuated inversion recovery (FLAIR) and T2-weighted images, in addition to bilaterally involving PPRF at the level of the pons, anterior to the cerebral aqueduct (Figs. 1-a, b). Two hyperintense lesions were also presented in the sagittal sections of the spinal MRI, one at the level of C7 and the other adjacent to T4. The lesions visible on the MRI were consistent with demyelination. After gadolinium injection, the PPRF lesions were not enhanced, however, T4 thoracic spine lesion was enhanced. Lumbar puncture was not performed because of patient’s dissent. Visual evoked potential (VEP) undertaken indicated no abnormality. Anti-aquaporin 4 (AQP4) and anti-myelin oligodendrocyte glycoprotein (MOG) antibodies were accomplished in order to rule out neuromyelitis optica (NMO) and both tests were negative. All other diagnostic tests and routine lab works were normal.

With respect to the clinical findings and the history of a previous attack, the patient has had two clinical attacks at two different sites in the CNS, which were confirmed using MRI, and an enhancing lesion at the level of T4, diagnosis of MS was made in terms of the 2017 revised McDonald’s criteria [4].

The patient was treated with 1000 mg of intravenous methylprednisolone for three consecutive days, along with other supportive measures. Eye motion indicated significant improvement by passing 1 week from therapy and complete ocular movements were retrieved on the one-month follow up visit of the patient.

## Discussion and conclusion

Complete bilateral horizontal gaze palsy is a rare clinical manifestation in neurological diseases [3]. Specific pathways (medial longitudinal fasciculus) and nuclei (abducens, oculomotor) are responsible for lateral gaze. Two sets of nerve fibers are originated from the abducens nuclei; one set of fibers result in abduction of the ipsilateral eye and the other set, termed internuclear neurons, travel through the medial longitudinal fasciculus (MLF) to the contralateral oculomotor nucleus, resulting in simultaneous adduction of the contralateral eye. Aside from the abducens and MLF fibers, the paramedian pontine reticular formation comprises multiple excitatory and inhibitory cell groups, which are arranged functionally in the reticular formation of the pons and medulla. Efferent connections from the PPRF exert their effects on the ipsilateral abducens nucleus, and therefore, their role in the initiation or suspension of conjugate horizontal eye movements [5]. Any lesions affecting the pathways and structures that were described can lead to abnormalities in conjugate ocular movements. Regardless of the type of the lesion, the clinical features that will be developed based on lesions’ site, will be briefly discussed.

Lesions affecting the abducens nuclei will cause ipsilateral horizontal gaze palsy, because of damage to motor neurons and internuclear neurons originating from the nuclei. Therefore, bilateral horizontal gaze palsy will be caused if lesion(s) involve both abducens nuclei. Vertical gaze will remain completely intact as these nuclei play no role in conjugate vertical eye movements. Conjugate horizontal gaze palsy can also be caused by lesion(s) involving both the abducens motor fibers and the MLFs preceding the abducens nucleus. Isolated damage to the MLFs will cause the classic INO where eye abduction is preserved, however, the ipsilateral eye will not be able to be adducted on conjugate horizontal gaze [6]. Lesions involving the PPRF bilaterally will also lead to bilateral horizontal gaze palsy similar to the involvement of bilateral abducens nuclei, as PPRF acts as a relay center that connects the frontal eye fields in the cerebral cortex to the abducens nuclei. As, rostral regions of the PPRF coordinate vertical saccadic movements [7], PPRF lesions may also result in decreased velocity of vertical eye movements, similar to our case.

Despite the fact that acute MS lesions usually enhance on the MRI after gadolinium injection [8], the PPRF lesions in our patient did not enhance. Although this does not completely rule out the acuteness of the lesion, it can be attributed to insufficient dosing of the contrast agent or inadequate timing.

An important differential diagnosis that should be discussed in further detail is neuromyelitis optica. NMO is an inflammatory disorder involving the central nervous system but distinct from MS based on clinical and imaging findings. NMO is mainly diagnosed through the detection of specific antibodies such as anti-AQP4 and anti-MOG antibodies. Although these antibodies were negative in our patient, the diagnosis of NMO cannot be completely ruled out as the diagnostic criteria for NMO have been described in patients with negative antibody panels [9]. Our patient did not have any evidence of optic neuritis neither on the MRI images obtained nor the clinical findings. Although the lesions shown in the images (Fig. 1-a, b) seem to be located near the area postrema, however our patient did not experience any signs of hiccups, nausea or vomiting pointing to area postrema syndrome. Patients with NMO can also present with acute myelitis similar to our patient. Nonetheless, spinal lesions in NMO must extend three or more vertebral segment longitudinally based on the criteria [9] unlike the spinal lesions of our patient which makes the diagnosis of NMO very unlikely.

Few case reports, describe patients with MS who have bilaterally experienced conjugate horizontal gaze palsy either as their first manifestation or along with their disease course.

Joseph et al. reports a case of a woman who was presented with unsteadiness and intermittent diplopia. Her symptoms progressed, so that she was unable to vertically and horizontally move her eyes. The nuclear magnetic resonance (NMR) image obtained from the patient suggested MS, and the lesion causing the symptoms was located at the level of the pons involving the ventral periaqueductal region. The author presumes that this lesion, which was located dorsal to the MLF region, caused the eye movement abnormalities and suggests that descending pathways for horizontal gaze control may be passing through the site [10].

In another case report [11], a patient was presented with limitation of eye movements to both sides and blurred vision. By applying magnetic resonance imaging, the patient was diagnosed with definite MS. The lesion causing the symptoms were located in the posteromedial part of the lower pontine tegmentum as T2-hyperintense lesions. Involvement of the abducens nuclei is considered the origin of conjugate gaze palsy in the patient.

Tan et al. describes a woman with MS who was also presented with bilateral horizontal gaze palsy, lack of convergence and left peripheral facial palsy. With respect to the location of demyelination in the brainstem of the patient’s MRI and the clinical manifestations, the author believed that bilateral PPRF involvement could be considered as the cause of the symptoms [7].

Ocular movement disturbances are common presentation in multiple sclerosis; however, bilateral horizontal gaze palsy is a rare clinical manifestation. Our patient presented with limitation of bilateral horizontal gaze. This presentation can also have various other etiologies such as infarct, vasculitis, and NMO. Therefore, a complete patient history including previous attacks, time frame of each symptom and other concurring systemic signs should be achieved from the patient. Laboratory workup should include complement levels, acute phase reactants, antineutrophil cytoplasmic antibodies (ANCA) and antinuclear antibodies (ANA) for vasculitis, and especially anti-NMO and anti-MOG antibodies to rule out NMO as a frequent mimicker of MS. MRI and other imaging techniques could also guide in the differentiation of demyelinating lesions from other types of lesions such as infarction. We assume that these lesions, probably involve the gaze centers in the pontine region bilaterally, and are causes of our patient’s clinical presentation. In a patient presenting with ocular movement abnormalities and gaze limitations, imaging should be obtained for locating suspected lesions in the pons, and multiple sclerosis anticipated, especially if presenting in a young or middle-aged female.

## Data Availability

Data sharing is not applicable to this article as no datasets were generated or analyzed during the current study.

## Abbreviations

ANA: Anti-Nuclear Antibody
ANCA: Antineutrophil Cytoplasmic Antibody
AQP4: Aquaporin 4
FLAIR: Fluid-Attenuated Inversion Recovery
INO: Internuclear Ophthalmoplegia
MLF: Medial Longitudinal Fasciculus
MOG: Myelin Oligodendrocyte Glycoprotein
MRI: Magnetic Resonance Imaging
MS: Multiple Sclerosis
NMO: Neuromyelitis Optica
NMR: Nuclear Magnetic Resonance
PPRF: Paramedian Pontine Reticular Formation
VEP: Visual Evoked Potential

## References

[1] Walsh RD, McClelland CM, Galetta SL (2012). The neuro-ophthalmology of multiple sclerosis. Future Neurol.

[2] Bradley WG (2004). Neurology in clinical practice: principles of diagnosis and management: Taylor & Francis.

[3] Hennerici M, Fromm C (1981). Isolated complete gaze palsy: an unusual ocular movement deficit probably due to a bilateral parapontine reticular formation (PPRF) lesion. Neuro-ophthalmology..

[4] Thompson AJ, Banwell BL, Barkhof F, Carroll WM, Coetzee T, Comi G (2018). Diagnosis of multiple sclerosis: 2017 revisions of the McDonald criteria. The Lancet Neurology.

[5] Amezcua L, Morrow MJ, Jirawuthiworavong GV (2015). Multiple sclerosis: review of eye movement disorders and update of disease-modifying therapies. Curr Opin Ophthalmol.

[6] Milea D, Napolitano M, Dechy H, Le Hoang P, Delattre J, Pierrot-Deseilligny C (2001). Complete bilateral horizontal gaze paralysis disclosing multiple sclerosis. J Neurol Neurosurg Psychiatry.

[7] Tan E, Kansu T (1990). Bilateral horizontal gaze palsy in multiple sclerosis. J Clin Neuro-ophthalmol.

[8] Olek MJ. Evaluation and diagnosis of multiple sclerosis in adults. In: Francisco Gonzalez-Scarano JFD, editor. Uptodate 2019.

[9] Wingerchuk DM, Banwell B, Bennett JL, Cabre P, Carroll W, Chitnis T (2015). International consensus diagnostic criteria for neuromyelitis optica spectrum disorders. Neurology..

[10] Joseph R, Pullicino P, Goldberg CDS, Rose FC (1985). Bilateral pontine gaze palsy: nuclear magnetic resonance findings in presumed multiple sclerosis. Arch Neurol.

[11] Kipfer S, Crook DW (2014). Isolated bilateral horizontal gaze palsy as first manifestation of multiple sclerosis. Mult Scler J.

